# Hearing loss and its association with the proteome of perilymph, cerebrospinal fluid, and tumor tissue in patients with vestibular schwannoma

**DOI:** 10.1038/s41598-024-64352-6

**Published:** 2024-06-19

**Authors:** Jesper Edvardsson Rasmussen, Peng Li, Göran Laurell, Jonas Bergquist, Per Olof Eriksson,

**Affiliations:** 1 Department of Surgical Sciences, Otorhinolaryngology and Head and Neck Surgery, Uppsala University, Uppsala, Sweden; 2 Computational Research Centre for Complex Chronic Diseases, Massachusetts General Hospital, Harvard Medical School, Boston, MA USA; 3 Department of Chemistry – BMC, Analytical Chemistry and Neurochemistry, Uppsala University, Uppsala, Sweden

**Keywords:** Hearing loss, Inner ear, Human, Vestibular schwannoma, Perilymph, Cerebrospinal fluid, Proteome, Differential gene expression, Pathway analysis, Complement cascade factor-H related protein 2, CFHR2, Inflammation, Cochlea, Inner ear, Neurodegeneration

## Abstract

This study examined the association between hearing loss in sporadic vestibular schwannoma patients and the proteome of perilymph (PL), cerebrospinal fluid (CSF), and vestibular schwannoma. Intraoperative sampling of PL and of CSF, and biopsy of vestibular schwannoma tissue, was performed in 32, 32, and 20 patients with vestibular schwannoma, respectively. Perilymph and CSF in three patients with meningioma and normal hearing were also sampled. The proteomes were identified by liquid chromatography coupled to high-resolution tandem mass spectrometry. Preoperative hearing function of the patients was evaluated with pure tone audiometry, with mean values at frequencies of 500, 1000, 2000, and 4000 Hz (PTA4) in the tumor-affected ear used to delineate three hearing groups. Analysis of the PL samples revealed significant upregulation of complement factor H-related protein 2 (CFHR2) in patients with severe to profound hearing loss after false discovery rate correction. Pathway analysis of biofunctions revealed higher activation scores in the severe/profound hearing loss group of leukocyte migration, viral infection, and migration of cells in PL. Upregulation of CFHR2 and activation of these pathways indicate chronic inflammation in the cochlea of vestibular schwannoma patients with severe to profound hearing loss compared with patients with normal hearing or mild hearing loss.

## Introduction

Hearing loss is a widespread global health problem, affecting one in five individuals^1^. Most individuals have mild hearing loss and struggle with conversations in noisy environments, but 5% of the global population are estimated to be in need of hearing rehabilitation interventions^1^. Hearing loss is not confined to the elderly population and impacts a significant portion of individuals under the age of 50, who comprise 38% of all cases^1^. Hearing loss elevates the risk of developing dementia^2^ and depression^3^, and is reported to double the risk of unemployment^4^ and adversely affect overall quality of life^1^. Despite these considerable health implications, the repertoire of pharmacological treatment options for hearing loss remains limited. One exception is corticosteroid use in clinical practice to treat idiopathic sudden hearing loss^5,6^ and vertigo in Ménière’s disease^7^.

Efforts to improve knowledge on human inner ear pathophysiology and to develop novel and efficacious therapies for hearing loss face many obstacles. Two major concerns are anatomic inaccessibility and inability to collect liquid and tissue samples from the inner ear, due to the substantial risk of inducing deafness. These difficulties also impede mapping of molecular properties of the human inner ear in vivo, which is imperative for development of novel therapies.

The human inner ear comprises three primary components, vestibular organs, cochlea, and endolymphatic sac. These components are interconnected by delicate channels and lined with membranes that partition the inner ear into two fluid-filled compartments, the perilymphatic and endolymphatic spaces. The perilymph is protected by a blood-perilymph barrier^8^, and has an ion composition similar to extracellular fluid^9^. The endolymph, which has a high potassium concentration^9^, is protected by the highly efficient blood-endolymph or intrastrial fluid- blood barrier^10^. The first description of the human perilymph (PL) proteome, published in 1988^11^, identified approximately 30 proteins. The knowledge base on the PL proteome has since been expanded through mass spectrometry-based studies, the first of which was published in 2011^12^. Understanding of the PL proteome has been further enhanced by investigations involving cochlear implant patients^13–16^ and vestibular schwannoma (VS) patients^17^. However, to the best of our knowledge, no previous study has examined associations between hearing loss and the proteomes of PL, CSF, and VS in samples from the same VS patient.

This study involved patients with sporadic VS or meningioma. Vestibular schwannomas originate from Schwann cells within the vestibular branch of the eighth cranial nerve (vestibulocochlear nerve). Meningiomas originate from the arachnoid^18^, the innermost layer of the meninges surrounding the brain. In most cases, meningiomas are benign and managed similarly to VS^19^.

While VS is a rare etiology of hearing loss, meningiomas are not typically considered to impact the inner ear and hearing. At the time of diagnosis, hearing function in VS patients exhibits substantial variations, with approximately one-third of patients presenting with normal hearing, one-third experiencing moderate hearing loss, and one-third having non-serviceable hearing^20^.

Sensorineural hearing loss in VS patients can be classified as cochlear, retrocochlear, or combined. A study on VS patients with profound hearing loss revealed that 33% had pure cochlear impairment, 13% had pure retrocochlear impairment, and 54% had combined impairment^21^. Mild hearing loss associated with cochlear impairment has been identified using distortion product otoacoustic emission measurements^22^. Histopathological quantifications show that reductions in spiral ganglia neurons, hair cells, stria vascularis, and the spiral ligament are correlated with decreased speech discrimination scores^23^. Retrocochlear impairment can be tested by measurement of auditory brainstem response, a cause of hearing loss in patients with VS according to a systematic review^24^. Intraoperative inner ear canal pressure measurements and auditory evoked potential assessments indicate a correlation between retrocochlear impairment and increased pressure from VS on the auditory nerve. The relationship between tumor size and hearing loss remains inconclusive^24^. Tumor growth is reported to be positively correlated with hearing loss progression, but only explains 8% of hearing loss progression^25^.

The diversity of preoperative hearing functionality in patients with VS enables studies of the association between hearing loss and proteome differences. Both VS and meningiomas can be treated by translabyrinthine removal of the tumor, which presents a unique opportunity to obtain liquid samples from the inner ear in vivo, and of cerebrospinal fluid (CSF) surrounding the tumor and tumor biopsies. Analysis of such samples could improve understanding of the underlying causes of hearing loss.

The aim of the present study was thus to assess the association between hearing loss and the proteomes of PL, CSF, and VS in patients with sporadic VS.

## Results

### Proteomes of PL, CSF, and VS

**Figure 1:**
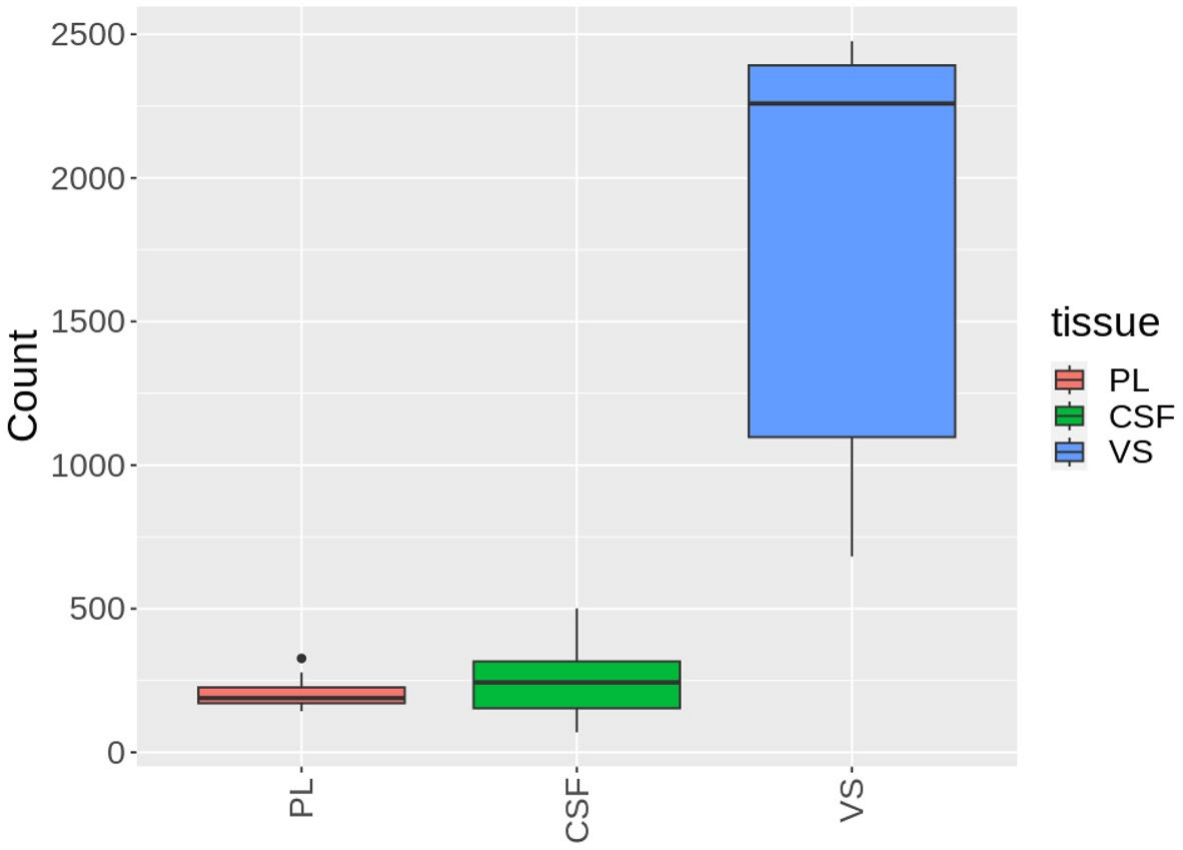
Box plot showing the number of different proteins identified in (**left**) perilymph (PL), (**center**) cerebrospinal fluid (CSF), and (**right**) vestibular schwannoma (VS) samples from VS patients. Boxes encompass the 1st–3rd quartiles and the median, while whiskers indicate the 1.5 interquartile range.

The proteomes of PL, CSF, and VS samples obtained from patients undergoing VS resection were first compared to describe the protein content. The median number of proteins in the PL, CSF, and VS samples was found to be 155, 201, and 2228, respectively. In total, 526 and 847 different proteins were identified in the liquid PL and CSF samples, respectively (n = 29) (Fig. 1). The total number of proteins identified in the VS samples (n = 20) was much larger (3201), due to its solid tissue nature (Fig. 1).

On assessing the number of patients in which each protein was identified, it was found that the proteome of the two fluids, PL and CSF, displayed an apparent variability characterized by a stable subset of proteins and a highly variable subset of proteins. The cutoff for the stable subset was set at identification in at least 20 out of 29 PL or CSF samples, while the cutoff for the highly variable subset was set at identification in one out of 29 PL or CSF samples. This yielded 126 and 114 proteins in the stable subset, and 155 and 215 proteins in the variable subset, for PL and CSF, respectively.

**Figure 2:**
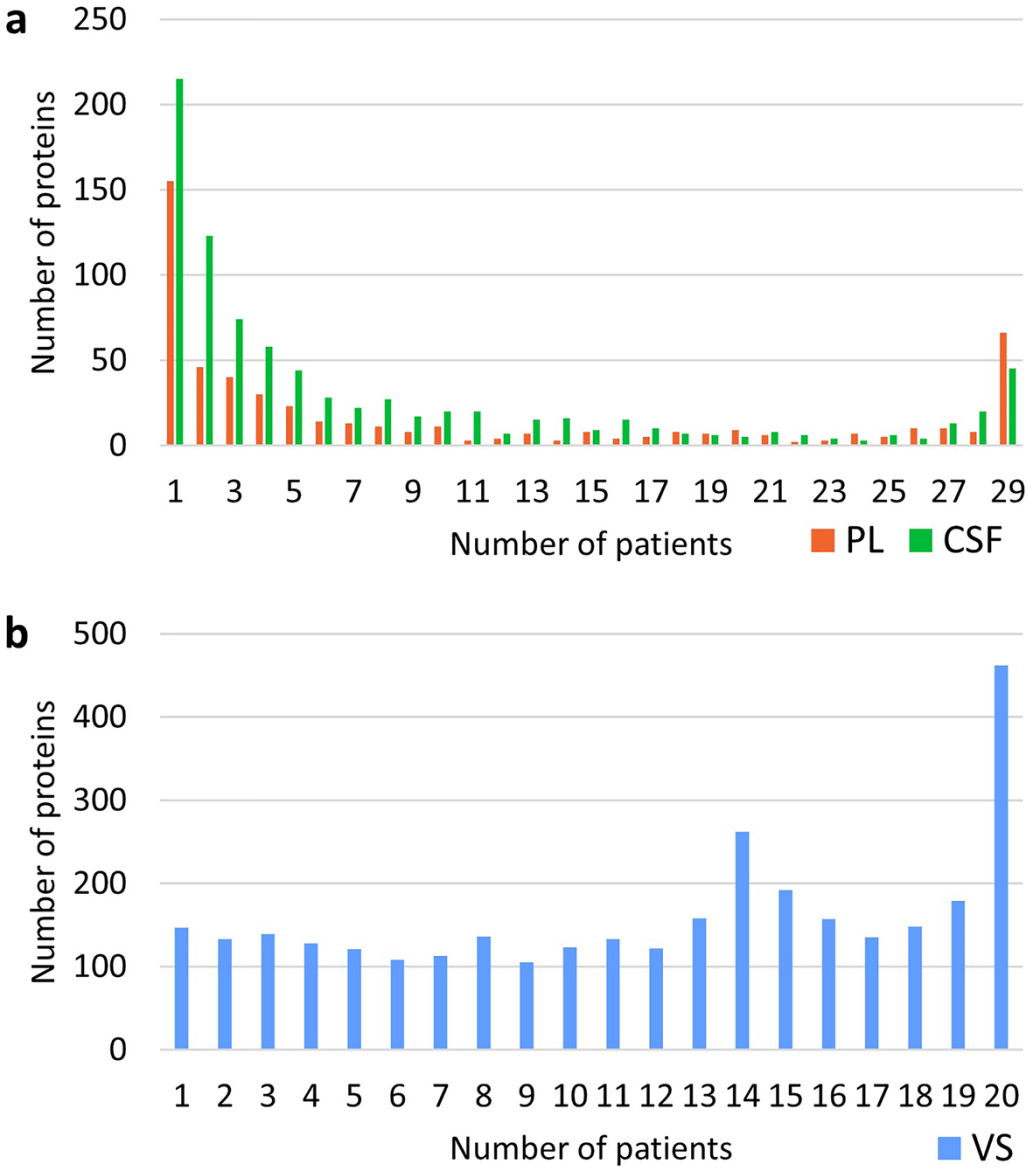
(**a**) Number of samples of perilymph (PL) (red) and cerebrospinal fluid (CSF) (green) from patients with vestibular schwannoma (VS) and (**b**) number of VS tissue biopsies in which each protein was identified. The histograms illustrate the distribution of proteins in bins, with the height of each bin indicating the number of proteins falling within the corresponding value range. For example, the bin at X = 29 in (a) represents 66 proteins found in all 29 perilymph samples, while the bin at X = 1 represents 155 proteins found only once among the perilymph samples.

Figure 2 shows the number of samples in which each protein was identified. In contrast to PL and CSF, the VS proteome exhibited less variability, with 1535 proteins identified in at least 14 out of 20 samples and 147 proteins identified only in a single sample. The PL proteome contained 33 proteins not identified in any CSF or VS sample.

As shown in Fig. 2a, 66 proteins were identified in all PL samples, among them cochlin, which was not identified in any of the CSF or VS samples from VS patients. Kallikrein-6 and amyloid-like protein 1 were only detected in CSF samples, and not in any of the PL or VS samples. Thirteen proteins were identified in at least 14 of the 29 CSF samples and not in any PL sample. The PL and CSF stable subsets had 96 proteins in common. Among the 3202 proteins identified in the VS samples, 2409 were not found in any of the PL or CSF samples. A complete table of the MaxQuant protein identification is provided in Supplement 1.

**Figure 3:**
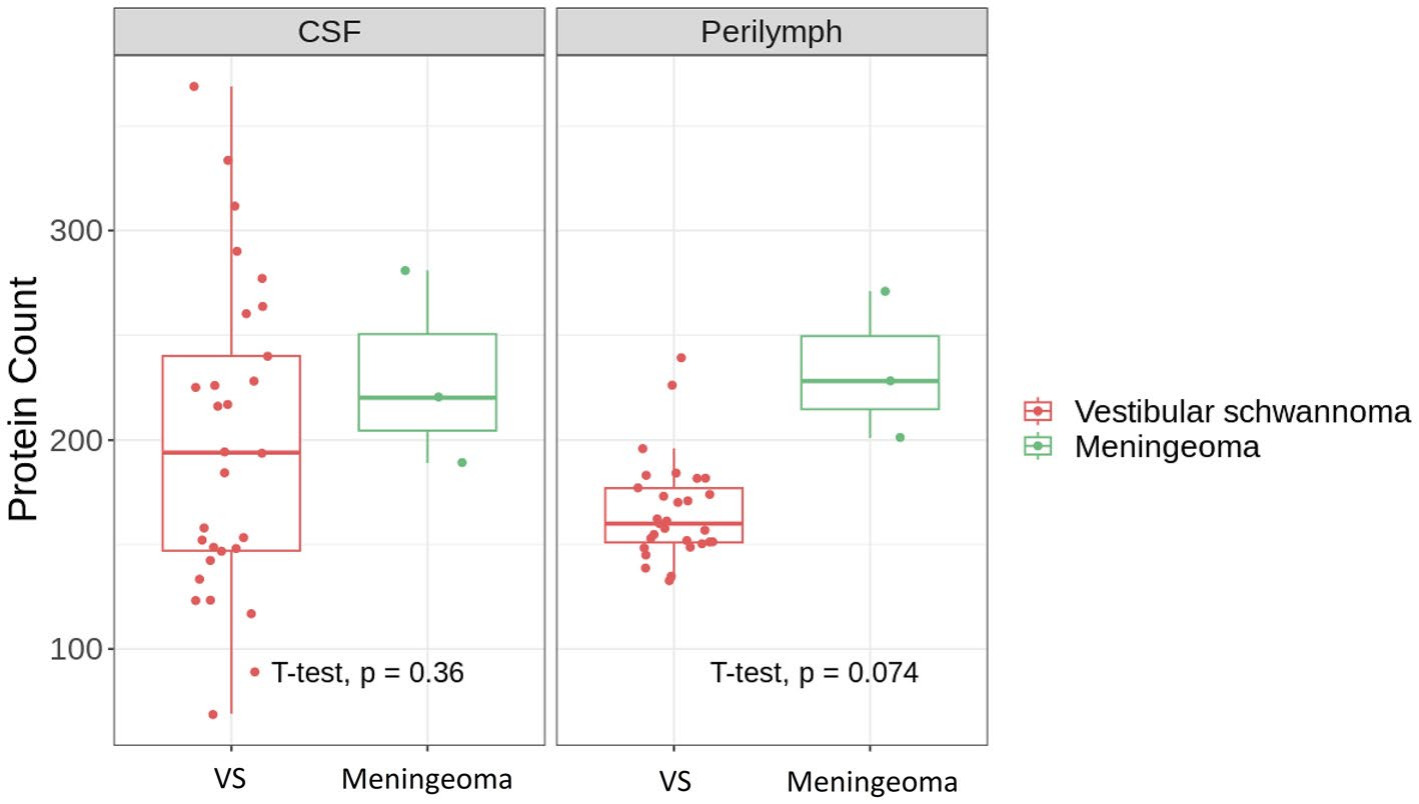
Box plot of number of proteins identified in perilymph and cerebrospinal fluid (CSF) samples from vestibular schwannoma patients (n = 29) and meningioma patients (n = 3). Boxes encompass the 1st–3rd quartiles and the median, while whiskers indicate the 1.5 interquartile range.

Comparison of numbers of proteins in PL and CSF samples from VS patients and meningioma patients revealed no significant differences (Fig. 3). However, there were only three samples in the meningioma group, so the power in statistical analysis was low. The total number of proteins identified in the three PL samples from meningioma patients was 411. Among these, 139 proteins were consistently present in all three samples, while 188 were identified once in the three samples. Notably, 64 out of the 139 proteins present in all three PL samples from meningioma patients were also found in all 29 PL samples from VS patients. However, among the 188 proteins identified in only one out of three meningioma samples, 105 were not detected in any of the PL samples from VS patients.

A total of 528 proteins were identified in the three CSF samples from meningioma patients. Of these, 89 were consistently present in all three samples, while 296 were detected once in the samples. Notably, 77 unique proteins were identified once in the CSF samples from meningioma patients and were not found in any of the VS patients. Additionally, two proteins were identified in two CSF samples from meningioma patients but were absent in the VS patients. These proteins were plasma membrane calcium-transporting ATPase 4 and guanine nucleotide-binding protein G subunit alpha.

### Association between hearing loss and patient characteristics

Multivariate linear regression of PTA4 of the VS-affected ear against clinical parameters revealed a correlation with sex, with males having higher degree of hearing loss (Pearson coefficient 0.64, *p* = 0.018). The other clinical parameters assessed (age, body mass index, comorbidity index, maximal extra meatal diameter, PTA4 of the contralateral ear) did not show any correlation with PTA4 of the VS-affected ear.

### Principal component analysis

Principal component analysis (PCA) was employed as an initial step to visualize all variations in the proteomic data and to identify clusters based on hearing groups 1–3 (representing normal hearing to severe/profound hearing loss)^26^.

**Figure 4:**
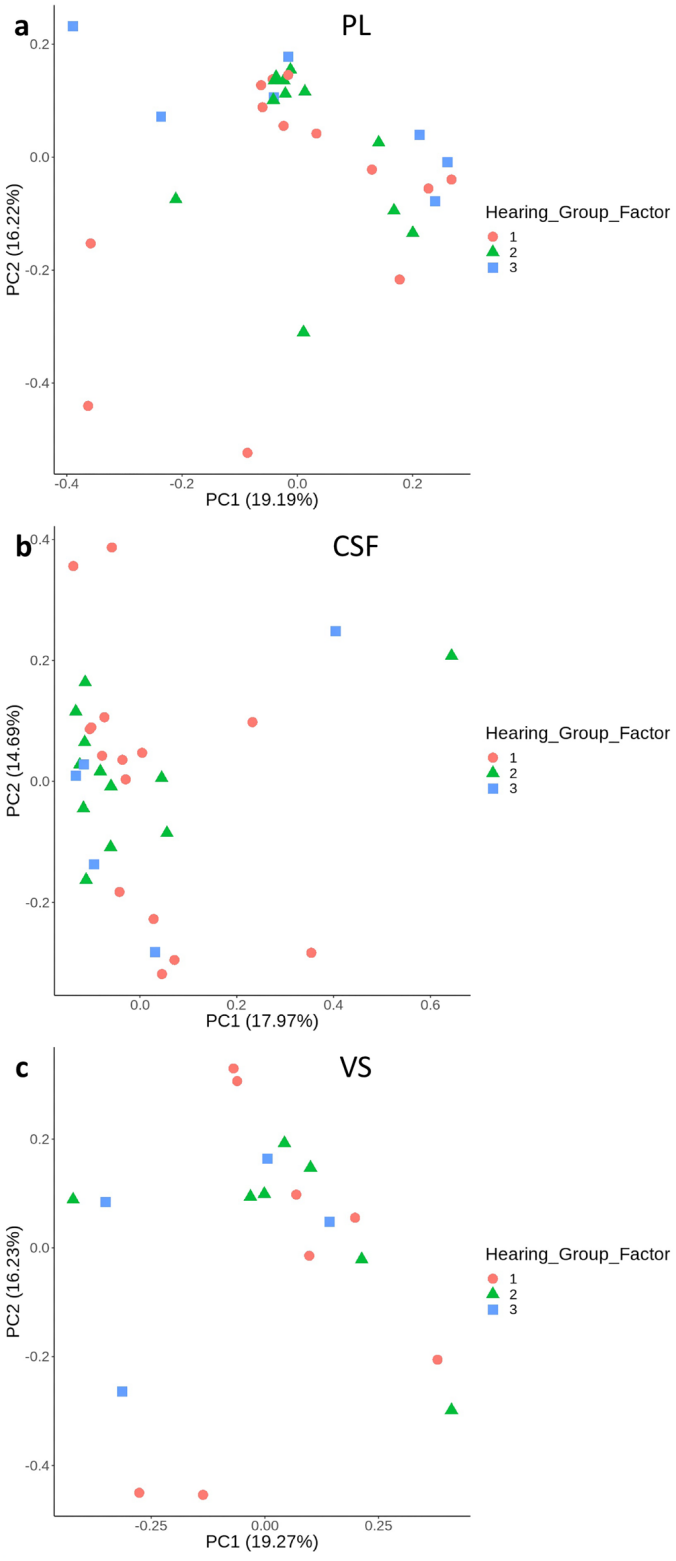
Principal component analysis (PCA) plots factored on hearing groups 1 (normal hearing to mild hearing loss), 2 (moderate to moderate severe hearing loss), and 3 (severe to profound hearing loss). In (**a**) perilymph (PL) and (**b**) cerebrospinal fluid samples from vestibular schwannoma (VS) patients (n = 28) and meningioma patients (n = 3) were included. In (**c)** VS tissue samples (VS patients n = 20) were included. PC1, PC2: principal components 1 and 2.

PCA was performed with separate plots for PL versus Hearing, CSF versus Hearing based on PL and CSF samples from both VS and meningioma patients, and for VS versus Hearing using only VS samples. (Fig. 4) One to two clusters emerged in these PCA plots, but with heterogenicity of hearing function.

### Differential expression analysis and pathway analysis

An in-depth analysis was performed on expression of individual proteins in PL, CSF, and tumor samples in relation to hearing loss in the tumor-affected ear. In differential expression analysis, two cutoffs were implemented to address the multiple testing problem by reducing the high number of tests to a more relevant subset of proteins. The first cutoff was a requirement of a minimum of 1.5-fold up- or downregulation of the proteins. The second cutoff was T-tests of differential expression exclusively conducted for proteins that met the first criterion, followed by false discovery rate (FDR) adjustment of *p*-values (q) using the Benjamin-Hochberg method^27^.

On applying an FDR cutoff of q ≤ 0.2, it was found that complement factor H-related protein 2 (CFHR2) was significantly upregulated in PL samples taken from hearing groups 3 and 2, compared with hearing group 1. Without FDR correction, nine proteins were found to be upregulated in PL samples from hearing group 3 compared with hearing group 1, while three proteins were downregulated (*p* ≤ 0.05). (Table 1) A detailed table is provided in Supplement 2.

**Table 1:** Number of differentially expressed proteins in samples from patients in the hearing groups 1 (normal hearing to mild hearing loss), 2 (moderate to moderate severe hearing loss), and 3 (severe to profound hearing loss).

	Downregulated	Upregulated
	(FC ≤ − 1.5)	(FC ≥ 1.5)
Hearing group 3 versus 1		
Perilymph	3	9
Cerebrospinal fluid	8	5
Vestibular schwannoma	33	23
Hearing group 2 versus 1		
Perilymph	0	6
Cerebrospinal fluid	4	3
Vestibular schwannoma	15	14
Hearing group 3 versus 2		
Perilymph	0	5
CSF	7	3
Vestibular schwannoma	35	32

These proteins could provide valuable insights into biological changes associated with hearing loss, although they did not pass the stringent FDR correction applied.

**Figure 5:**
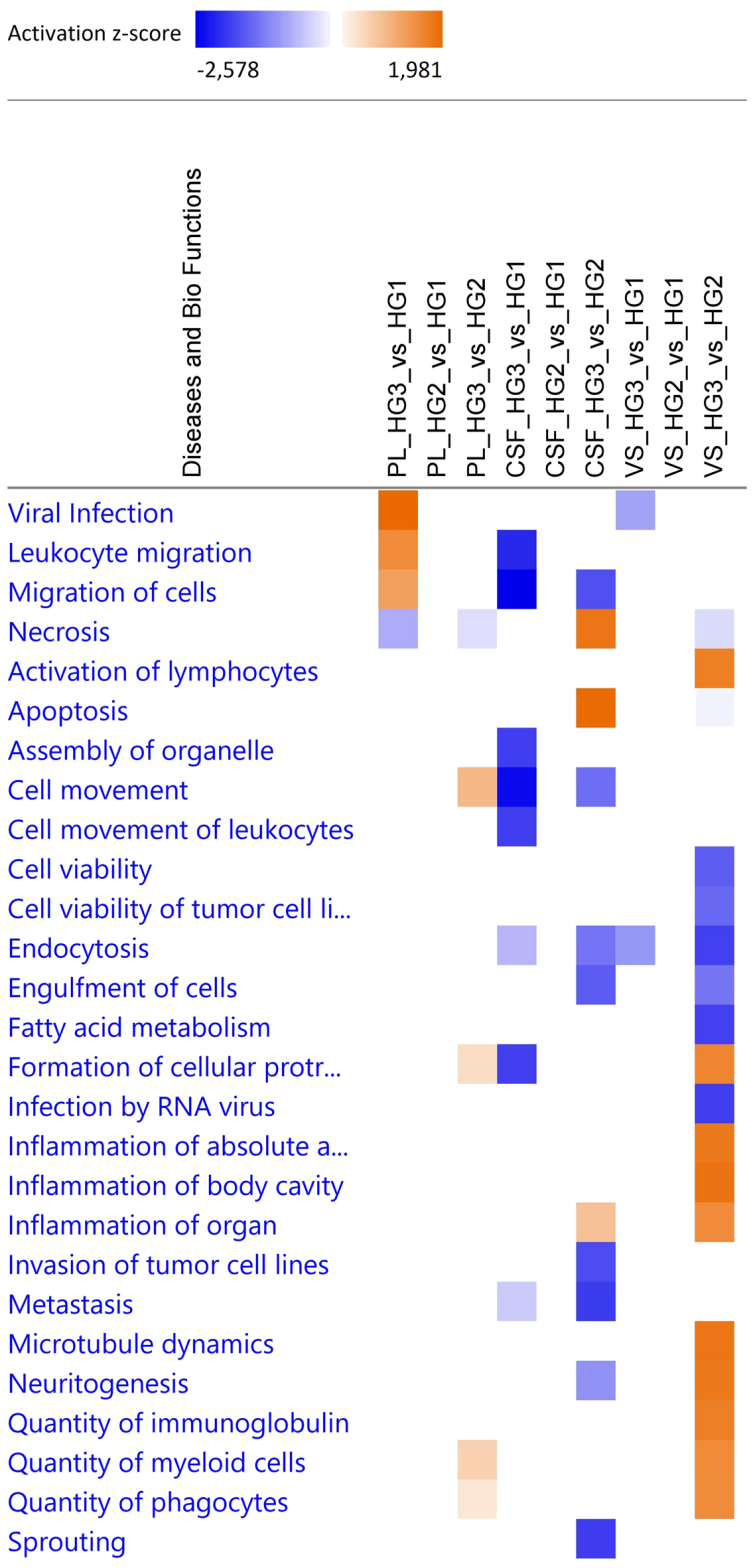
Disease and biofunction pathways, analyzed with Ingenuity Pathway Analysis software and presented with activation z-score, of differentially expressed proteins in vestibular schwannoma (VS), perilymph (PL), and cerebrospinal fluid (CSF) samples mapped for hearing groups 1 (normal hearing to mild hearing loss), 2 (moderate to moderate severe hearing loss), and 3 (severe to profound hearing loss). Pathways were filtered based on Benjamini–Hochberg adjusted *p*-value < 0.05 and z-score > 1.

Pathway analysis was conducted to better understand the functions of the proteins identified in differential expression analysis. The proteins were organized into functional hearing groups (referred to as pathways). When comparing two groups, pathway activation was calculated as down- or upregulated^28^. However, the PL and CSF samples had a limited number of proteins meeting the criteria for inclusion in pathway analysis, thereby restricting the number of pathways that could be compared. The results of pathway analysis for the differentially expressed proteins in PL, CSF, and VS samples from the three hearing groups are presented in terms of disease and biofunction in Fig. 5.

Comparison of results for PL samples from hearing group 3 and hearing group 1 revealed higher pathway activation scores for leukocyte migration, migration of cells, and viral infection, and a lower score for necrosis, in group 3 samples. In CSF samples, activation score for necrosis and apoptosis was higher in hearing group 3 than hearing group 2 samples, while activation scores for leukocyte migration and migration of cells were lower in samples from hearing group 3 compared with hearing group 1. In VS samples, hearing group 3 had no category with higher activation score compared with group 1, but higher activation scores for seven pathways related to immune system activity compared with group 2. Lower activation scores in hearing group 3 compared with hearing group 2 were seen for six pathways, notably in cell viability.

## Discussion

Since the first mass spectroscopy-based proteome study in 2011^12^, the human perilymph proteome has been further explored in a series of studies on samples obtained during surgery^13,16,17,29^. While the number of patients in each study has been limited, the results have improved knowledge of the PL proteome profile and its function under normal and pathological conditions. The present study provides novel information regarding the association between the PL, CSF, and VS proteomes and hearing loss in the tumor-affected ear in patients with sporadic VS.

The overall aim of the study was to identify proteins that can distinguish between patients with varying degrees of hearing loss in the ear affected by the tumor. Differential analyses of the proteomes in PL, CSF, and tumor samples, followed by FDR correction, revealed strong upregulation of CFHR2 (40.3-fold change) in the PL of patients with severe to profound hearing loss (hearing group 3) compared with patients with normal hearing or mild hearing loss (hearing group 1). CFHR2 is part of the regulation of complement activation, competing with the complement cascade inhibitor complement Factor H in the fluid phase of complement activation, thereby increasing complement activation^30^. The pronounced upregulation observed in the present study suggests that CFHR2 has its effect in PL in the inner ear. CFHR2 is part of the CFHR-Factor H gene cluster and located downstream of Factor H^31^. The complement Factor H-related gene family includes five genes (*CFHR1*-*CFHR5*)^31^. Increases in *CFHR1-CFHR5* gene copy numbers or dimerization have been linked to the pathogenesis of the kidney diseases C3 glomerulopathy and atypical hemolytic uremic syndrome^32^. Additionally, CFHR2 and CFHR3 are reported to be associated with a risk of cardiovascular disease in children with chronic kidney disease^33^ and kidney function decrease in IgA nephritis^34^. High-activity, low-frequency variants of the *CFHR2* allele are reported to be associated with a sensory deficit, namely age-related macular degeneration^35^. Based on the results obtained in this study, it can be speculated that the upregulation of CFHR2 in the cochlea has a decremental impact on hearing due to dysregulation of complement activation, leading to inflammation in the inner ear.

All PL samples contained cochlin, but it was absent in CSF and VS samples. Cochlin has been well mapped in the inner ear^36–38^, and cochlin mutations are a known genetic cause of adult late-onset sensorineural hearing loss. However, homozygous missense cochlin mutation causes prelingual deafness^39^. Cochlin mutations can alter the structure of the spiral ligament^40^ and cochlin may promote innate immunity inflammation^41^. The CSF samples also contained unique proteins compared with the PL samples, such as amyloid-like protein, which is associated with disease of the central nervous system^42^. The specific differences between the PL and CSF proteomes clearly demonstrate that PL origin is separate from that of CSF.

The PCA results on total variation in the proteomes in relation to hearing loss revealed only one small cluster among the VS tissue biopsies. This low association between the VS proteome and hearing loss could have several explanations. First, the variations in the proteomes related to hearing function were not sufficiently large to affect the principal components 1 and 2, which explained the largest proportion of the variation in the dataset. Second, the low sample size may have affected the power of the statistical analysis. Third, the limited association between the VS proteome and hearing loss is a reliable finding. However, the low number of samples in the observed cluster limited detailed exploration of this cluster, and it was not pursued.

A recent study on long-term follow-up of untreated VS patients revealed that 40% maintained serviceable hearing 10 years later^20^. Hearing loss in VS patients has been categorized previously as pure cochlear impairment in 33%, pure retrocochlear impairment in 13%, and combined impairment in 54%^21^. Histopathological examinations conducted on human temporal bones have revealed that cochlea affected by VS exhibits degeneration of the cochlear nerve ganglion, leading to a reduction in axons to the inner and outer hair cells, as well as loss of stereocilia^43,44^. In another study, profound hearing loss in VS patients was found to be associated with degeneration of the stria vascularis and the spiral ligament^23^. The stria vascularis is critical for hearing function, as it generates the requisite electrical potential for the sensory function of hair cells^45–47^. It has been suggested that presence of pure cochlear impairment indicates an impact of VS tumor on the microenvironment within the cochlea, even with preserved nerve function. Tests in recent experimental and clinical studies show that tumor necrosis factor α (TNFα), a pro-inflammatory cytokine^48^, may be associated with pure cochlear hearing loss^49,50^. The finding in the present study of an association between hearing loss and high upregulation of CFHR2 in PL further confirms that cochlear impairment among patients with sporadic VS could be caused by chronic inflammation.

Knowledge of the immune environment within the cochlea has been extended in recent years. In the normal mammalian cochlea, a heterogenous mixture of B-cells, T-cells, macrophages, natural killer cells, and neutrophils has been identified^51^. Studies of the human cochlea have detected macrophages^52–54^, CD4 and CD8 T-cells^55^, and B-cells^56^ in various cochlear compartments. To study the immune response, noise trauma and infection experimental models have primarily been employed in mice and rats. Noise trauma is reported to increase the presence of immune cells^57^, with macrophages and supporting cells, e.g., the pillar cells, playing a role in clearing damaged cells^58^. Although immune cells are reported to play a key role in the immune defense of the cochlea, the regulation of immune response is poorly understood.

Recent studies on PL samples from cochlear implant patients have reported a lower concentration of VEGF-D and higher concentrations of IL-9 and IL-13 among patients with no residual hearing compared with those with residual hearing^15^. Upregulation of IGHV1-2, IGHV1-46, and IGKV6-21, indicating B-cell involvement, is reported to be associated with poor hearing performance. In contrast, IGKV4-1 and Complement C8 upregulation has been shown to be associated with excellent hearing performance one year after cochlear implantation^16^. Complement C8 contributes to the membrane attack complex binding to C5b^59^ and cell lysis^32^. Another effect in the complement cascade is activation of C3a and C5a, promoted by the *CFHR* gene family, leading to adaptive immune response by cell recruitment and inflammation^32^. Our finding of CFHR2 upregulation and higher activation score for leukocyte migration in PL suggests that an adaptive immune response could be a mechanism contributing to hearing loss. A more detailed understanding of the complexity of the inner ear immune response is needed to understand the possible duality in immune-mediated protective and negative effects on cochlear structures.

A recent MRI study on VS patients found that degree of hearing loss was related to intensity of T1 gadolinium enhancement in the labyrinth^60^. Increased gadolinium enhancement in the labyrinth has also been demonstrated in an experimental study on the effects of furosemide on blood-perilymph and blood-endolymph barrier function^61^. Therefore, the high activity of CFHR2 in PL in VS patients with severe to profound hearing loss could be related to altered barrier integrity, allowing more macromolecules and cell migration over the blood-perilymph barrier.

We identified several proteins in PL, CSF, and VS samples that displayed significant differential expression, but did not meet the stringent Benjamin-Hochberg FDR correction criteria. Nevertheless, as this study involved in vivo samples from patients, we believe these proteins may offer valuable insights into changes in biological functions in the VS-affected cochlea. Among patients with severe to profound hearing loss, activation scores were higher for the pathway’s leukocyte migration, migration of cells, and viral infection in PL. In VS tissue from patients with severe to profound hearing loss, there was an increase in activation of leukocytes, quantity of immunoglobulin, quantity of myeloid cells, and quantity of phagocytes compared with patients with moderate hearing loss. These pathways suggest an elevated adaptive immune response in both PL and VS.

In CSF, in contrast to PL, the activation score of leukocyte migration and migration of cells was lower in samples from patients with severe to profound hearing loss than in patients with normal or mild hearing loss. This may indicate that the heightened inflammatory activity in the inner ear, possibly induced by the tumor, is not mediated through changes in CSF.

In terms of limitations of the present work, it is important to note that conducting studies on the human inner ear in vivo poses significant challenges. The low number of patients included in the present study limited the power of the statistical analysis. However, VS is a rare etiological factor in hearing loss and few patients need treatment by translabyrinthine surgery, so it is difficult to gather samples from large cohorts of patients with VS. Another limitation is the variability in the time course of hearing loss among the VS patients and the time interval between onset of disease and treatment, which for most patients are unknown. The majority of patients with VS show an increase in pure-tone thresholds and decreased speech discrimination score during wait-and-scan treatment^62^. Speech discrimination score is a more qualitative measure of sensory function than the pure-tone threshold and adds valuable information in hearing assessments in a rehabilitation situation^63^. However, speech discrimination tests were only available for 15 of the patients in the present study and were therefore not included, limiting the analysis of this aspect of hearing function. A strength of the study was the inclusion of three tissue types, facilitating comparisons of changes in the proteomes related to hearing function in PL, VS, and CSF.

## Conclusions

The findings obtained in this study underscore the potential role of tumor-induced inflammation processes within the cochlea as a mechanism of sensorineural hearing loss. CFHR2 levels were higher in perilymph obtained from patients with severe to profound hearing loss than in perilymph samples from patients with normal hearing or mild hearing loss. Pathway analysis revealed higher activation of the pathways viral infection, leukocyte migration, and migration of cells in PL, all related to immune response, in patients with severe to profound hearing loss. These findings suggest that complement cascade-mediated inflammation in the cochlea could be a mechanism of sensorineural hearing loss in patients with sporadic VS.

## Methods

### Study participants

Patients with radiologically diagnosed sporadic VS or sporadic meningioma and scheduled for translabyrinthine surgery at Uppsala University Hospital were included in the study after providing signed informed consent. The indications for surgery at our center are growing tumors, multicystic tumors, or larger tumors compressing the brainstem or cerebellum. The exclusion criteria were prior neurosurgery, radiation therapy, neurological disease, and multimorbidity. In total, 36 patients were included in the study. Post-operatively, all tumors were confirmed by histopathological examination to be vestibular schwannoma WHO grade 1 or benign meningioma. Patient characteristics are presented in Table 2. One patient was excluded after sample collection due to previous radiation therapy. Sixteen of the 35 patients were female and 19 were male, and 32 had VS and three had meningioma (Table 2). Median age was 50 years (range 17–72 years). Only four patients had one point on Charlson/Quan comorbidity index^64^, as prior to inclusion all patients were deemed medically fit for a major neurosurgical intervention.

**Table 2:** Characteristics of patients included in the study.

Sex	Age (years)	Tumor diameter(mm)^a^	PTA4 tumor ear^b^	PTA4 contra-lateral ear^b^	Difference in PTA4^b^	Hearing group	Tumor type
F	52	25	5	5	0	1	Meningioma
F	34	30	9	8	1	1	VS
F	45	18	9	9	0	1	VS
M	45	31	10	10	0	1	VS
M	37	17	13	5	8	1	VS
F	44	32	15	1	14	1	Meningioma
F	42	18	15	5	10	1	VS
M	57	33	15	10	5	1	Meningioma
F	55	19	16	1	15	1	VS
M	41	44	19	3	16	1	VS
M	26	19	24	4	20	1	VS
F	55	40	26	18	8	1	VS
M	49	18	26	6	20	1	VS
F	50	6	29	19	10	1	VS
F	35	25	30	11	19	1	VS
F	56	20	34	4	30	1	VS
M	45	37	35	5	30	2	VS
F	35	27	36	8	28	2	VS
M	17	20	48	3	45	2	VS
F	65	19	50	38	12	2	VS
M	55	24	51	39	12	2	VS
F	62	37	51	11	40	2	VS
M	29	25	52	5	47	2	VS
M	31	27	58	10	48	2	VS
M	41	40	61	33	28	2	VS
F	59	30	61	8	53	2	VS
M	55	27	62	5	57	2	VS
F	71	31	63	44	19	2	VS
F	64	16	73	21	52	3	VS
M	46	13	79	13	66	3	VS
M	61	23	81	8	73	3	VS
M	53	22	83	38	45	3	VS
M	72	28	84	30	54	3	VS
M	60	11	110	9	101	3	VS
M	65	27	x	x	x	x	VS

^a^Tumour diameter defined as maximal extrameatal diameter.

^b^Pure tone average of 0.5 kHz, 1 kHz, 2 kHz, and 4 kHz.

Hearing function was assessed with pure-tone audiometry prior to surgery, using audiometers that were calibrated according to International Standards Organization (ISO) criteria. Pure-tone average 4 (PTA4) was calculated as the mean of 500 Hz, 1000 Hz, 2000 Hz, and 4000 Hz. PTA4 threshold values for the hearing loss definitions established by Stevens et al.^65^ were used to divide the patients into three hearing groups. Patients in hearing group 1 had normal hearing or mild hearing loss (PTA4 < 35), those in hearing group 2 had moderate or moderate-severe hearing loss (PTA4 35–64), and those in hearing group 3 had severe to profound hearing loss (PTA4 > 64). One patient did not have preoperative pure-tone audiometry and was not included in differential expression analysis of the proteomes in relation to hearing. Hearing group 1 consisted of 16 patients with a median difference in PTA4 between the tumor-affected ear and contralateral ear of 10 dBHL (range 0–30 dBHL). Hearing group 2 consisted of 12 patients with a median difference in PTA4 of 35 dBHL (range 12–57 dBHL). Hearing group 3 consisted of 6 patients with a median difference in PTA4 of 60 dBHL (range 45–101 dBHL).

Linear regression analysis was conducted on the hearing groups, with sex, age, BMI, co-morbidity index, maximal extramental tumor diameter, and PTA4 of contralateral ear as predictors, using the Finalfit package in R. This approach facilitated comprehensive assessment of the relationships between these predictors and the hearing outcomes under study.

Samples were collected during translabyrinthine surgery after thorough hemostasis and cleaning of the surgical field to avoid contamination. PL samples were collected after an incision of the round window with a sharp needle. PL was aspirated into a sterile tip with an Eppendorf Research®-Plus Variable pipette (5–10 μL, Eppendorf AG, Hamburg, Germany) to a maximum of 10 μL, over a period of approximately 10 s. CSF samples were collected in the cerebellopontine angle when the dura mater was opened to expose the tumor. The samples were immediately placed in liquid nitrogen and then stored in a freezer at -80ºC. The samples were assessed for blood contamination visually by color and by quality in mass spectrometry analysis. Three PL samples and three CSF samples were discarded because of contamination, resulting in 32 PL samples and 32 CSF samples being included. VS tissue samples were not collected at the beginning of the study, but later added to the scope of the study, which resulted in 20 VS tissue biopsies. Table 2 including sample id and tissue type can be found in Supplement 3.

### Mass spectrometry of PL and CSF samples

A 3 μL aliquot of PL or CSF sample was mixed with 20 μL of digestion buffer containing 6 M urea and 50 mM ammonium bicarbonate, and sonicated in sonication bath for 3 min. Then 50 μL of 50 mM ammonium bicarbonate were added to each sample before digestion.

The proteins were reduced, alkylated, in-solution digested by trypsin, and desalted with C18 ZipTips according to standard operating procedures. The peptide filtrate collected was vacuum-centrifuged to dryness using a Speedvac system. Each sample was then dissolved in 21 µL 0.1% formic acid and further diluted 15 times, prior to liquid chromatography tandem mass spectrometry (LC–MS/MS) analysis.

The resulting peptides were separated in reversed-phase on a C18-column, applying a 90 min long gradient, and electro-sprayed on-line to a QEx-Orbitrap mass spectrometer (Thermo Finnigan). Tandem mass spectrometry was performed applying higher-energy collisional dissociation (HCD).

### Mass spectrometry of tumor tissue samples

Subsamples of approximately 25 mg of tumor tissue were homogenized in 600 μL of lysis buffer (1% β-octyl-glucoside, 6 M urea, protease inhibitor cocktail in PBS) for 10 × 1 s using a sonication probe (3 mm probe, pulse 1 s, amplitude 30%). After homogenization, the samples were incubated for 60 min at 4 °C under mild agitation. The tissue lysates were clarified by centrifugation for 10 min at 16000 × g and 4 °C.

Total protein concentration in the samples was then measured using the DC Protein Assay, with bovine serum albumin (BSA) as standard. Subsamples corresponding to 50 µg protein were taken out for digestion.

The proteins were reduced, alkylated, and on-filter digested by trypsin using 3 kDa centrifugal filters (Millipore, Ireland) according to standard operating procedures. The peptide filtrate collected was vacuum-centrifuged to dryness using a Speedvac system. Each sample was then dissolved in 150 µL 0.1% formic acid and further diluted five times prior to LC–MS/MS analysis. The peptides were separated in reversed-phase on a C18-column with 150 min gradient and electro-sprayed on-line to a Q Exactive Plus mass spectrometer (Thermo Finnigan). Tandem mass spectrometry was performed applying HCD.

### Database search and protein quantification

The raw data (in the form of RAW files) were subjected to processing by the MaxQuant software, version 2.2.0.0, with the built-in Andromeda search engine spearheading the database searches. The MS/MS spectra correlations were drawn against a FASTA database that housed proteins specifically from *Homo sapiens*. These proteins were sourced from the Uniprot database (release date June 2022). To gauge the identification FDR, a decoy search database encompassing common contaminants along with a reverse database was employed. A 1% FDR was deemed acceptable in this study. The search parameters deployed were as follows:

- Error tolerance was set at a maximum of 10 ppm for the survey scan and 0.6 Da for the MS/MS analysis.
- Trypsin was selected for enzyme specificity.
- A maximum of one missed cleavage site was accepted.
- For variable modifications, N-terminal protein acetylation and methionine oxidation were chosen.
- Carbamidomethylation of cysteine residues was predetermined as a fixed modification.

For quantification, label-free procedures were conducted using the conventional LFQ settings (Stabilize large LFQ ratios: True, Require MS/MS for LFQ comparisons: True, iBAQ: False). The average area derived from the three most abundant peptides for any matched protein was employed to determine protein abundances, as proposed by Silva et al.^66^. The LFQ intensities were transformed by the log 2 function. Quantifiable proteins were scrutinized based on the following criteria: a minimum of two peptides covered and identification in at least three samples.

### Principal component analysis

Post-quantification, to acquire a broad overview of data variance and detect potential clusters or outliers among the samples, PCA was conducted using R software. PCA is a statistical method that reduces data dimensionality while preserving essential information by transforming the data into new variables known as principal components^26^. These components capture variation, with the first principal component (PC1) explaining the highest proportion of the variability, followed by subsequent components^26^. The PRCOMP function was employed to execute the PCA on data that were both centered and scaled. The first two principal components (PC) were then visualized using the ggplot2 package^67^. Each point in the PCA plot symbolizes a sample, and the spatial distance between points portrays their similarities based on their proteomic profiles. Sample groups are differentiated using distinct colors and shapes.

### Differential expression analysis and pathway analysis

To determine the significance of alterations among multiple contrasts, a moderated t-test was executed using the LIMMA package version 3.14^68^. Normalization of data was achieved utilizing the quantile normalization approach across all proteins. The process of identifying differentially expressed proteins incorporated blocking of the patient subject ID, to ensure a smooth comparison between distinct patient cohorts. The subsequent *p*-values underwent correction via the Benjamini–Hochberg algorithm^27^, with significant determinations based on an adjusted *p*-value < 0.05 and absolute fold change > 1.5 for the provided contrast.

Pathway analysis was performed using the Ingenuity Pathway Analysis by QIAGEN^69^, adhering to its default parameters. In brief, pathway analysis uses data on protein abundance and differential expression within a sample and annotates proteins with information regarding their established functions, subcellular localization, and interactions, sourced from a comprehensive database. The input data were the proteins detected as significant (*p* < 0.05, absolute fold change > 1.5). Filtering of significant pathways was determined using Benjamini–Hochberg adjusted *p*-value < 0.05 and z-score > 1.

### Ethical statement

The study was approved by the local ethical review committee in Uppsala (reference 2013/255) and the amendment was approved by the Swedish ethical review authority (reference 2021–03,433). Oral and written informed consent was obtained from all study participants prior to inclusion. The study adhered to the terms of the Helsinki declaration.

### Supplementary Information

Supplementary Information 1.

Supplementary Information 2.

Supplementary Information 3.

## Data Availability

Data is provided within the manuscript or supplementary information files.
